# Biochemical changes in patients during hypothyroid phase after thyroidectomy

**DOI:** 10.25122/jml-2021-0297

**Published:** 2022-01

**Authors:** Ghusoon Al-Janabi, Hayder Neamah Hassan, Ali Al-Fahham

**Affiliations:** 1. Clinical Laboratories Department, Applied Medical Sciences College, Karbala University, Kerbala, Iraq; 2. Faculty of Medicine, University of Kufa, Kufa, Iraq; 3. Faculty of Nursing, University of Kufa, Kufa, Iraq

**Keywords:** thyroidectomy, hypothyroidism, lipid profile, liver enzymes

## Abstract

Hypothyroidism is the most common long-term consequence after total thyroidectomy. The objective of the present study was to evaluate the lipid profile and liver function in patients after hypothyroidism. Sixty patients who underwent a surgical operation to remove thyroid were included in this study, and thirty healthy subjects were used as a control. The study was conducted at Al-Sadr Medical City in Al-Najaf city, in Iraq, from October 2020 to March 2021. Thyroid-stimulating hormone (TSH) was very high in patients at a hypothyroid stage after hypothyroidism. The results showed a significant increase in total cholesterol, triglycerides (TG), low-density lipoprotein (LDL), and the ratio of total cholesterol/high-density lipoprotein (HDL). The study also revealed a significant increase in the liver enzymes aspartate aminotransferase (AST) and alanine transaminase (ALT) and a significant decrease in alkaline phosphatase (ALP) in patients with thyroidectomy compared to the control group. The correlation matrix revealed a strong positive correlation between TSH levels and total cholesterol, triglycerides, LDL, AST, ALT, and ALP. It was concluded that hypothyroidism, the major consequence of thyroidectomy, causes dysfunction in lipid metabolism and liver enzymes resulting in secondary hyperlipidemia and liver dysfunction.

## Introduction

Thyroid hormones are necessary for the normal development, growth, and function of organs. Thyroid hormones affect hepatic function by controlling the metabolic rate of all cells, including hepatocytes; the liver metabolizes thyroid hormones and helps regulate their systemic endocrine effects. Thyroid problems have been related to several metabolic imbalances in the body [1]. Thyroidectomy is a well-established technique for thyroid gland removal. It is a frequent operation in contemporary medicine and may be used to treat cancer, benign illness, or hormonal disorder resistant to medicinal treatment [2]. Thyroid hormone increases cholesterol production in the liver by inducing 3-hydroxy-3-methylglutaryl coenzyme A (HMG-CoA) reductase activity. Cholesterol production rises in hyperthyroidism, although total blood cholesterol and low-density lipoprotein cholesterol (LDL-C) concentrations drop due to enhanced LDL-C metabolism [3]. Numerous factors contribute to accelerated atherosclerosis in hypothyroid individuals, including insulin resistance, hypercholesterolemia, enhanced low-density lipoprotein (LDL) oxidation, hypercoagulable condition, and increased peripheral vascular resistance. Thyroid hormones have a direct vasodilatory impact on smooth muscle cells in the vascular system [4]. Acute hypothyroidism is a major consequence of thyroidectomy before thyroid hormone replacement therapy is achieved [5]. It was found that liver function may be affected by acute hypothyroidism resulting from thyroidectomy. While the consequences of hyperthyroidism on the liver are widely documented, those of hypothyroidism are less well known. Because thyroxine and triiodothyronine are critical for maintaining a normal basal metabolic rate, Bohinc *et al.* found that when the liver is injured, the hepatic stromal cells react by down-regulating deiodinases, resulting in hypothyroidism. This response is mediated via the Hedgehog pathway [6]. Clinically, hypothyroidism may resemble the signs and symptoms of chronic liver illness, such as myxedema ascites, myopathy, and even myxedema coma [7]. The goal of this study is to evaluate lipid profile, which includes cholesterol, low-density lipoproteins (LDL), high-density lipoproteins (HDL), and very-low-density lipoproteins (VLDL), as well as liver function, which includes aspartate transaminase (AST), alanine transaminase (ALA), and alkaline phosphatase (ALP) in hypothyroid patients.

## Material and Methods

Sixty patients who underwent a surgical operation to remove thyroid glands were included in this study, and thirty healthy subjects were used as a control. The study was conducted at Al-Sadr Medical City in Al-Najaf, Iraq, between October 2020 and March 2021. Venous blood samples were drawn after a 12–14h overnight fast in the hypothyroid phase (three weeks after surgical removal of thyroids). Biochemical measurements for lipid profile and liver enzymes were made using an auto-analyzer (Hitachi Modular System; Mannheim, Germany).

## Results

**Figure 1.**
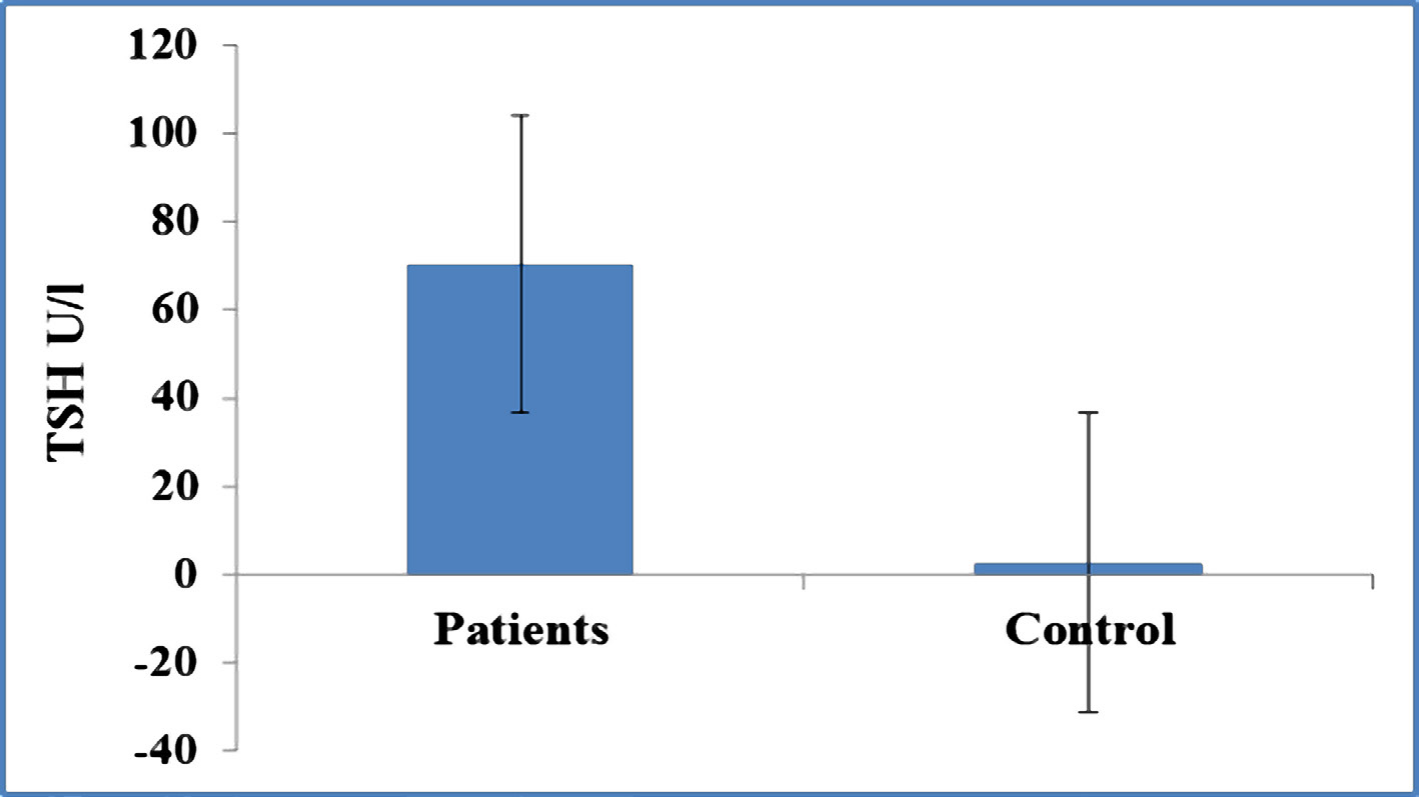
Differences in TSH (U/l) between patients and control groups.

Thyroid-stimulating hormone (TSH) was measured in the control and patient groups, and it was expected to be very high in patients in the hypothyroid stage after hypothyroidism (Figure 1). Table 1 shows the characteristics of the patients and control groups. There was no significant difference between patients and control groups regarding age, gender, and body mass index (BMI). Regarding lipid profile between patients and control groups, the current study showed a significant increase in total cholesterol, TG, LDL, and the ratio of total cholesterol/HDL (Table 2). Table 3 reveals the differences in liver enzymes between patients and control groups. There was a significant increase in the liver enzymes AST and ALT and a significant decrease in ALP in patients with thyroidectomy compared to the control group. The correlation matrix revealed a strong positive correlation between the serum levels of TSH and total cholesterol, triglycerides, LDL, AST, ALT, and a negative correlation with ALP (Table 4).

**Table 1.** Characteristics of the patients and control groups.

**Items**		**Patients**		**Control**		**Chi-Square**	**P-value**
		**Freq**	**%**	**Freq**	**%**		
**Age**	**18–24**	22	36.67	10	33.33	0.63	0.72
	**25–31**	19	31.67	8	26.67		
	**32–38**	19	31.67	12	40.00		
**Gender**	**Male**	39	65	16	53.33	1.15	0.28
	**Female**	21	35	14	46.67		
**BMI**	**Normal**	37	61.67	16	53.33	1.78	0.41
	**Overweight**	14	23.33	6	20.00		
	**Obese**	9	15	8	26.67		

**Table 2.** Differences in lipid profile between patients and control groups.

**Items**	**Patients**	**Control**	**T-Test**	**P-value**
	**Mean±SD**	**Mean±SD**		
**Total Cholesterol**	311.2±23.2	225.5±33.1	9.33	0.000
**Triglycerides**	165.7±16.5	151.2±14.6	4.24	0.000
**LDL**	210.2±34.1	146.3±32.8	5.26	0.000
**HDL**	42.7±5.6	43.7±6.4	0.49	0.12
**VLDL**	36.4±3.4	34.2±5.9	0.65	0.24
**Total Cholesterol/HDL**	8.7±1.1	6.4±2.3	2.94	0.03

**Table 3.** Differences in liver enzymes between patients and control groups.

**Items**	**Patients**	**Control**	**T-Test**	**P-value**
	**Mean±SD**	**Mean±SD**		
**AST**	41.12±13.22	21.12±12.37	6.91	0.000
**ALT**	42.6±8.26	21.6±8.33	7.12	0.000
**ALP**	56.78±18.82	79.78±19.34	5.23	0.000

**Table 4.** Correlation between TSH and studied markers.

**Items**	**Pearson correlation coefficient (r)**	**P-value**
**Total Cholesterol**	0.415	0.000
**Triglycerides**	0.526	0.000
**LDL**	0.356	0.000
**HDL**	0.184	0.17
**VLDL**	0.217	0.24
**Total Cholesterol/HDL**	0.123	0.23
**AST**	0.541	0.000
**ALT**	0.432	0.001
**ALP**	-0.356	0.02

## Discussion

Permanent hypoparathyroidism is the most often seen long-term consequence after total thyroidectomy, and it may result in substantial morbidity and higher expenditures. Its prevalence ranges from 30% to 60% [8]. Secondary dyslipidemia has been linked to several thyroid disorders, and prior study has shown that acute hypothyroidism after thyroid surgery has a negative effect on lipid profile and endothelial function. Increased TSH levels may be due to other variables such as lipoprotein (a), insulin sensitivity indices, or homocysteine, rather than a direct influence of TSH on flow-mediated dilation (FMD). On the other hand, increased TSH levels may directly affect endothelial and vascular function. A recent study showed that TSH promotes the production of cyclic 3, 5-adenosine monophosphate in human coronary artery smooth muscle cells, suggesting that it may operate directly on these cells [9]. Liu *et al.* conducted a systematic review and included sixteen observational studies and found that levels of total cholesterol (TC), LDL-C, and TG levels were significantly elevated in clients with subclinical hypothyroidism (SH) in comparison to euthyroidism, indicating a correlation between SH and increased lipid profile [10]. Although lower thyroid function is associated with decreased HMG-CoA reductase activity, individuals with overt hypothyroidism have higher TC and LDL-C values. This is related to reduced LDL-receptor activation, which results in decreased LDL and IDL catabolism [11]. Additionally, overt hypothyroidism is associated with a reduction in lipoprotein lipase (LPL) activity, which impairs the clearance of TG-rich lipoproteins. As a result, overtly hypothyroid individuals may also present with high TG levels linked with increased VLDL and, on occasion, fasting chylomicronemia. In hypothyroidism, the VLDL and IDL particles are high in cholesterol and apolipoprotein E, mimicking the -VLDL particles seen in type III hyperlipoproteinemia. There are studies demonstrating no significant changes in sdLDL levels between overt hypothyroid patients and healthy controls [12]. Another research examined the impact of overt hypothyroidism on LDL sub-fractions in the short term. Patients with thyroidectomy (n=28) were taken off replacement medication 2–3 weeks before radioactive iodine ablation. Patients had a rise in LDL-C, mainly attributable to an increase in big LDL particles, while sdLDL remained stable [13]. TSH was shown to be strongly associated with lipid components even when measured within its reference range, irrespective of FT3, TT3, FT4, and TT4. These correlations suggest that even in clinically euthyroid people, small changes in TSH may lead to elevated lipid levels and the incidence of hypercholesterolemia [14]. Similarly, significant associations between TSH and lipid profiles have been discovered in euthyroid Chinese, Korean, Latin American, and Spanish populations. However, in these investigations, only conventional serum lipid confounding variables such as age, gender, BMI, and smoking status were included [15]. It is well established that the blood lipid levels of obviously hypothyroid patients are invariably greater than those of healthy controls. Subclinical hypothyroidism is linked with increased TC and LDL-C levels, and in some studies, also with increased TG and reduced HDL-C levels. Recent investigations have shown strong associations between FT3 and TG and HDL-C; TT3 and TC, LDL-C and TG; and FT4 and TC, LDL-C, HDL-C, and TG in euthyroid individuals [16]. Consistent with these findings, the present research discovered substantial positive relationships between TSH levels and total cholesterol and low-density lipoprotein cholesterol levels using a correlation matrix.

These findings all lead to the same conclusion: blood thyroid hormone levels have an evident effect on serum lipid levels, even within the normal range. Thus, except for gender, age, BMI, and other well-established confounding factors, thyroid hormones may play a significant role in the connection between TSH and lipid markers. It is critical to assess TSH’s impact on lipid profiles regardless of thyroid hormone level. If these critical factors affecting lipid levels are not considered, the connection between TSH and lipid profile seems dubious. We established a substantial connection between TSH and lipid markers in this research even after adjusting for thyroid hormone effects [17]. Throughout the normal and high TSH ranges, we found a significant positive connection between serum TSH, ALT, and AST activities, as well as a similar inverse correlation between FT4 and serum liver enzyme activity concentrations. Consequently, individuals with TSH >4.5 mU/l had a significantly greater frequency of elevated serum ALT or AST activity concentrations than those with TSH <0.1 mU/l. Notably, despite controlling for a broad range of potential confounding factors, such as age, gender, fasting glucose, and lipid characteristics, these results remained mostly unchanged [15]. The impact of TSH on TC levels was discovered to be a combination of direct and indirect effects through thyroid hormones. Our findings clearly demonstrate that thyroid hormones play a critical role in the relationship between TSH and lipids and that TSH may also play an independent function [14]. Regarding alkaline phosphatase, the results of the following study revealed that serum ALP levels are significantly low (P<0.001) in the hypothyroid patients’ group as compared to the control. The declining level of serum ALP among hypothyroid patients in the current study agrees with the finding of Mane and Bhagwat [18] and Al-Hindawi *et al.* [19], which mentioned that ALP values in hypothyroid were significantly lower compared to control. Another study made by Pandey *et al.* [20] showed a significant increase in serum ALP level in hypothyroidism. The reduction in blood ALP activity in hypothyroid individuals may be due to low serum magnesium and zinc levels; restoring serum magnesium and zinc levels to normal also restored serum ALP activity to normal; or it may be due to reduced ALP synthesis by osteoblasts, which needed thyroid hormones.

## Conclusion

It was concluded that hypothyroidism, the major consequence of thyroidectomy, causes dysfunction in lipid metabolism and liver enzymes resulting in secondary hyperlipidemia and liver dysfunction.

## Acknowledgments

### Conflict of interest

The authors declare no conflict of interest.

### Ethical approval

This case-control study was approved by the medical ethics committee from the Faculty of Medicine/Kufa University (Reference no.: MEC-13 on April 21/2020).

### Consent to participate

Written informed consent was obtained from all participants and parents of patients younger than 18 years.

### Personal thanks

The authors want to offer special thanks to Dr. Karrar Abdulzahra for his support.

### Authorship

GAJ contributed to data collection, manuscript concept, laboratory analysis, and revision. HNH contributed to data collection and analysis. AAF is the corresponding author and contributed to data collection, manuscript concept, writing, laboratory analysis, submission of the manuscript, and galley proof.
